# Do Patients with Parkinson’s Disease Benefit from Dynamic Body Weight Support? A Pilot Study on the Emerging Role of Rysen

**DOI:** 10.3390/biomedicines11082148

**Published:** 2023-07-30

**Authors:** Laura Ciatto, Massimo Pullia, Graziana Tavilla, Biagio Dauccio, Daniela Messina, Maria Cristina De Cola, Angelo Quartarone, Roberta Cellini, Mirjam Bonanno, Rocco Salvatore Calabrò

**Affiliations:** IRCCS Centro Neurolesi “Bonino-Pulejo”, Via Palermo, C.da Casazza, S.S 113, 98123 Messina, Italy; laura.ciatto@irccsme.it (L.C.); massimo.pullia@irccsme.it (M.P.); graziana.tavilla@irccsme.it (G.T.); biagio.dauccio@irccsme.it (B.D.); daniela.messina@irccsme.it (D.M.); mariacristina.decola@irccsme.it (M.C.D.C.); angelo.quartarone@irccsme.it (A.Q.); roberta.cellini@irccsme.it (R.C.); roccos.calabro@irccsme.it (R.S.C.)

**Keywords:** Parkinson’s disease, neurorehabilitation, postural stability, body weight support system, Rysen system

## Abstract

Parkinson’s disease (PD) is a neurodegenerative disorder characterized by motor and non-motor alterations. Typical motor symptoms include resting tremors, bradykinesia (hypokinesia or akinesia), muscular stiffness, gait alterations, and postural instability. In this context, neurorehabilitation may have a pivotal role in slowing the progression of PD, using both conventional and innovative rehabilitation approaches. Thirty patients (15 males and 15 females) affected by PD were enrolled in our study. We randomly divided the patients into two groups, an experimental group (EG) and a control group (CG). In particular, the EG performed gait and balance training using the Rysen system, which is an innovative body weight support (BWS) system, whilst the CG received conventional physiotherapy. Both groups underwent 20 sessions, five times weekly, with each session lasting about 40 min. At the end of the training sessions (T1), we found that both groups (EG and CG) achieved clinical improvements, although the EG showed better scores for post-treatment regarding global motor functioning and postural stability compared to the CG. In conclusion, our results suggest that the Rysen system, which is an innovative BWS tool, could be considered a valid device for improving postural control and global motor functions, when compared to conventional gait training, in patients affected by PD.

## 1. Introduction

Parkinson’s disease (PD) is a progressive neurodegenerative disorder caused by the loss of dopaminergic neurons in the substantia nigra (midbrain) [1]. PD is characterized by motor and non-motor symptoms. Among the motor concerns, the most common include resting tremors, bradykinesia (hypokinesia or akinesia), muscular stiffness, and postural instability (PI) [2]. In particular, PI can be observed in the early stages of the disease, but it tends to worsen during the disease progression, exposing PD patients to a higher risk of falls and imbalances and potentially leading to fractures and hospitalizations [3]. PD patients also show gait disturbances (short stride length, low gait speed, high stride variability, and increased double support time) associated with freezing and festination [4]. Moreover, non-motor manifestations, such as orthostatic hypotension and psychological and behavioural problems, further reduce the patients’ quality of life [5]. In this context, motor rehabilitation is fundamental to maintaining voluntary motility and coordination, reducing muscle stiffness, and containing PI. This, in turn, improves independence during the activity of daily living. Generally, the conventional physiotherapy approaches for PD include gait training with a treadmill to increase gait speed and endurance, core exercises to improve balance reactions and postural stability, and hydrotherapy to reduce muscle stiffness and improve gait function [6]. Moreover, combined physiotherapy exercise training (including aerobic, resistance, and balance training) has shown beneficial effects not only for balance, muscle strength, gait recovery, and endurance, but also for slowing the progression of motor impairments [7]. Unconventional physiotherapy approaches (such as dance exercise therapy, Tai Chi, Qigong, and yoga) may also be considered in the treatment of PD patients [8]. In particular, dance exercise therapy is useful for promoting balance recovery through stepping and turning in different directions, shifting the centre of mass, and coordinating lower limb movements with the upper limbs [9,10]. Moreover, recent technological developments allow the implementation of innovative devices, such as treadmills equipped with virtual reality (VR) systems, that further promote motor recovery, boosting neuroplastic processes through high-intensity training, several repetitions, and increased motivation [11]. Additionally, body weight support (BWS) systems have been widely used in the context of motor neurorehabilitation. According to Atan et al. [12], a body-weight-supported treadmill training program with 10% or 20% support improved walking distance, balance, the Unified Parkinson’s Disease Rating Scale (UPDRS) motor score, quality of life, and fatigue compared with unsupported treadmill training in subjects with PD. Among BWS systems, the Rysen system combines horizontal and vertical assistive forces through complete 3D BWS, providing a safe environment to train balance and gait functions [13]. In fact, according to a systematic review and metanalysis, the use of BWS in PD could promote the alignment and straightening of the trunk, thus improving posture, which is fundamental to the recovery of static and dynamic balance in this patient population [14]. 

The aim of this pilot study was to investigate the role of the Rysen system in improving balance, postural stability, and functional status in a sample of PD patients.

## 2. Materials and Methods

### 2.1. Study Design 

In this pilot study, we enrolled thirty patients affected by PD, who were divided randomly into two groups (experimental group—EG and control group—CG) using a web-based app (www.randomization.com) (accessed on 1 January 2023) for block randomization (block size = 4). Both groups were evaluated before (T0) and after (T1) training rehabilitation sessions with a specific motor assessment battery. The EG underwent rehabilitation sessions using the Rysen system, while the CG received conventional rehabilitation sessions, with only the manual guidance of a physiotherapist. The experimental training consisted of 20 sessions, 5 times weekly, with each session lasting about 40 min, whereas patients in the CG received the same amount of overground training.

### 2.2. Study Population and Setting

The 30 PD patients (15 females and 15 males), with a mean age of 66.36 ± 8.28, were enrolled at the PD neurorehabilitation unit of the IRCCS Neurolesi “Bonino-Pulejo” (Messina, Italy) between October 2021 and December 2022.

Patients were included if they: (1) had a diagnosis of PD according to the Movement Disorder Society Clinical Diagnostic Criteria for Parkinson’s Disease; (2) were aged 50 to 70 years; (3) had moderate to advanced disease (Hoehn and Yahr classification grade 2 ≤ 4); and (5) were able to walk independently. Participants were excluded if they: (1) had cognitive, visual, or auditory deficits that could impair the comprehension and/or execution of the proposed exercise; (2) had associated comorbidities that prevented upright posture and walking (e.g., hypotension); (3) refused consent or were unable to provide informed consent; (4) had contraindications to the use of the technological instrumentation provided for the dynamic path of movement, including a weight > 135 kg or a height > 200 cm; or (7) had open lesions or bandages in the area of contact with the harness. 

All experiments were conducted according to the ethical policies and procedures approved by the local ethics committee (IRCCS-ME-45/2020). All participants gave their written informed consent. The clinical and demographic characteristics are summarized in Table 1.

### 2.3. Procedures 

Of the thirty PD patients enrolled in this pilot study, fifteen patients underwent the experimental program using the Rysen system (EG), whereas the other fifteen were submitted to conventional rehabilitation treatments (CG). Both groups received the same amount of training (i.e., 20 sessions, 5 times weekly, with each session lasting about 40 min), but two different methods were used (experimental/Rysen vs. conventional). 

#### 2.3.1. The Rysen System 

**Figure 1:**
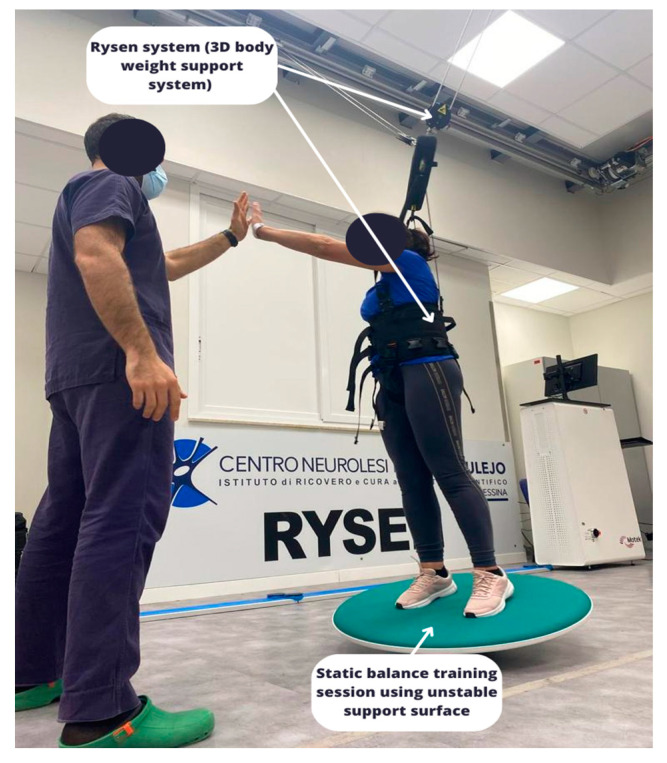
Image showing a physiotherapist performing a balance training session with the Rysen system.

Patients enrolled in the EG performed the same amount of training as the CG, but on the Rysen system (Motek, The Netherlands), which is a 3D BWS system designed to promote the functional recovery of walking by enhancing balance reactions. Notably, the Rysen system allows the patient’s natural gait pattern and active movements to be maintained in a large and safe workspace. The safety system consists of a harness fixed to a swing bar by means of four weight lock buckles, which protect the patient from falls [13] (Figure 1).

Physiotherapists can personalize the vertical and horizontal assistive forces matched with a specific BWS for each patient by performing training sessions focused on active participation, learning by mistake, and minimizing the risks of falls (see Table 2 for a detailed description of EG exercise training). 

**Figure 2:**
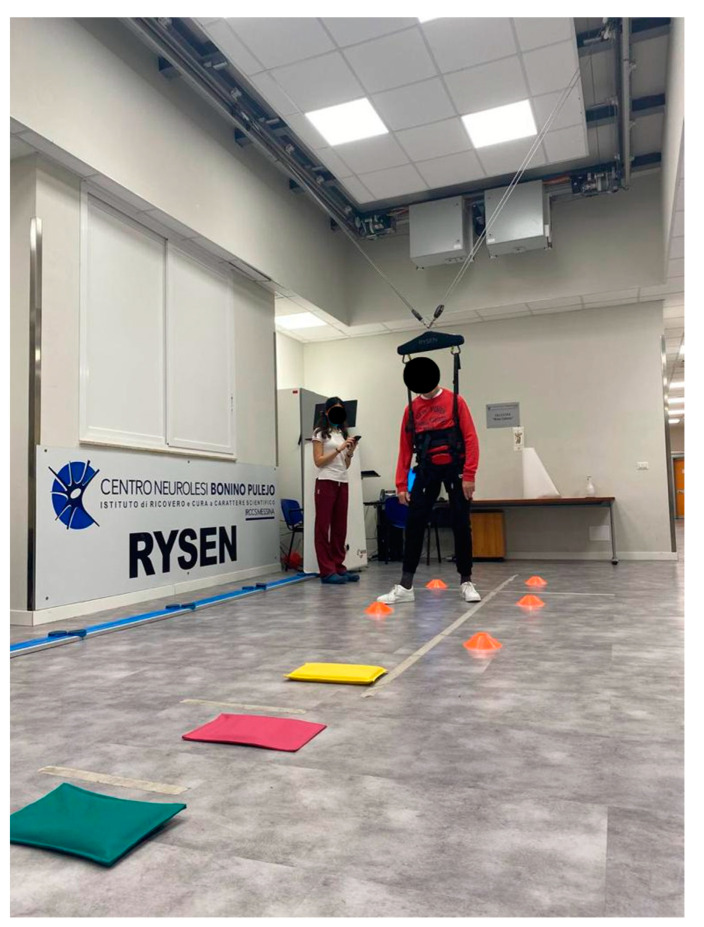
Image showing a PD patient during a Rysen gait training session.

The Rysen system ensures different exercise modes by simulating various terrains, real-life walking conditions, and everyday activities. The exercise modes that were used to train the PD patients include the following:

- Stand up: this mode was used to initiate a training session by gently bringing the individual into a standing position in the longitudinal direction of the room, in order to perform a postural alignment with the aid of the BWS.
- Walking: when the walking mode was selected, all kinds of gait exercises **were** performed by editing the vertical and horizontal supports (see Figure 2).
- Static and dynamic balance: the subject shifted his weight from side to side to find the equilibrium position. There were three-dimensional virtual boundaries within which the subject tried to keep his balance. When the subject leaned beyond one of the boundaries, the support increased. Editable parameters: vertical support (see Figure 1).
- Stairs: virtual boundaries on both sides of the subject in the longitudinal direction of the room were used to simulate stairs. Thanks to lateral resistance, the subject was supported to maintain the walking position, reducing the difficulty of lateral balance. The therapist can personalize the vertical and horizontal supports.
- Sit down: this was used at the end of a training session to return the individual to a sitting position, and as an exercise during the training session.

#### 2.3.2. Conventional Rehabilitation Treatment

Patients enrolled in the CG underwent a conventional rehabilitation program (with each session lasting about 40 min) that included the following motor activities: overcoming obstacles, tandem and slalom walking, gait training, sit-to-stand exercises to improve core stability, weight-shifting exercises in an upright position, and monopodal and bipodal balance exercises. The physical exercises for patients in the control group aimed to train their postural control, walking ability, and static and dynamic balance. All exercises were quite similar to those performed by patients in the EG, with different degrees of variability related to the patients’ needs and capabilities. During all sessions, the patients were supervised by the physiotherapists to prevent falls (a detailed description of CG exercise training is reported in Table 3). 

### 2.4. Outcome Measures 

Outcome measures were recorded for both groups before the rehabilitation sessions (T0) and after the rehabilitation sessions (T1), by two skilled physiotherapists (L.C. and B.D.) that were blind to the treatment allocation. They administered the following outcome measures: (i) The Berg Balance Scale (BBS) [15] was used to assess static and dynamic balance within 14 tasks (a score of 41–56 indicates a low fall risk, 21–40 indicates a medium fall risk, and 0–20 indicates a high fall risk). (ii) The Falls Efficacy Scale—International (FES-I) [16] measured the level of concern relating to falls for 16 different conditions during daily life. The score ranges from 1 (not at all) to 4 (maximum fear). A score between 7 and 8 indicates a low worry, between 9 and 12 indicates a moderate concern, and between 14 and 28 indicates a high perception of falling. (iii) The Tinetti Scale (POMA) [17] consists of 16 items, with seven related to gait and nine related to balance. Performance on all items is scored from 0 to 1 or 2 for a maximum score of 28, with a higher score indicating better gait and balance. A total score of 19 or less indicates a high risk for falling and a score between 19 and 24 indicates a moderate risk. (iv) The Movement Disorder Society—Unified Parkinson’s Disease Rating Scale, section III (MDS-UPDRS-III) [18] was used for the characterization of PD progression; it includes items regarding upper and lower extremity bradykinesia, rigidity, and, to a lesser extent, some midline functions (facial expression, speech, gait, and posture). (v) The Functional Independence Measure (FIM) [19] was administered to evaluate global functioning, and it comprises 18 items with a total score ranging from 0 to 126. The test includes six subscales: self-care, sphincter control, transfer, locomotion, communication, and social cognition ability. A higher score indicates less disability for basic daily functions. 

### 2.5. Statistical Analysis

The data were analysed using R version 4.3.0 (Vienna, Austria) [20], considering a *p* < 0.05 as statistically significant. A non-parametric analysis was performed. Hence, a one-tailed Mann–Whitney U Test and Fisher’s Exact Test were used to compare the two groups at baseline/follow-up, when appropriate. We performed an analysis of covariance (ANCOVA) in order to assess whether the type of treatment influenced the clinical outcome independently from the score difference at baseline. 

Notably, each model had, as the dependent variable, the test score of the clinical outcome measure at T1; as the independent variable, the categorical variable representing the “group”: 1 = experimental and 0 = control; and, as a covariate, the test score of the clinical outcome measure at baseline (T0). In this way, the adopted model allowed us to follow the changes at T1 for both groups, regardless of whether the two groups (EG and CG) at T0 were similar or not. We performed ANOVA to verify whether the interaction term “Group * test score at T0” was significant for the model. 

## 3. Results

No significant differences at baseline between the clinical assessment scores of the two groups were found, except for the POMA (*p* = 0.032). On the contrary, at the follow-up, the two groups differed in their BBS (*p* = 0.032), UPDRS (*p* = 0.019), and FIM (*p* = 0.005) scores.

The ANCOVA assumptions were always satisfied. Since the interaction term “Group * test score at T0” was not significant, it was not considered in the ANCOVA model-fitting. The results of this analysis showed that the effect of both treatments involved an improvement to the patients’ test scores, although it was significantly different for UPDRS (*t* = −2.20; *p* < 0.05) and FIM (*t* = 3.67; *p* < 0.01), as reported in Table 3. 

**Figure 3:**
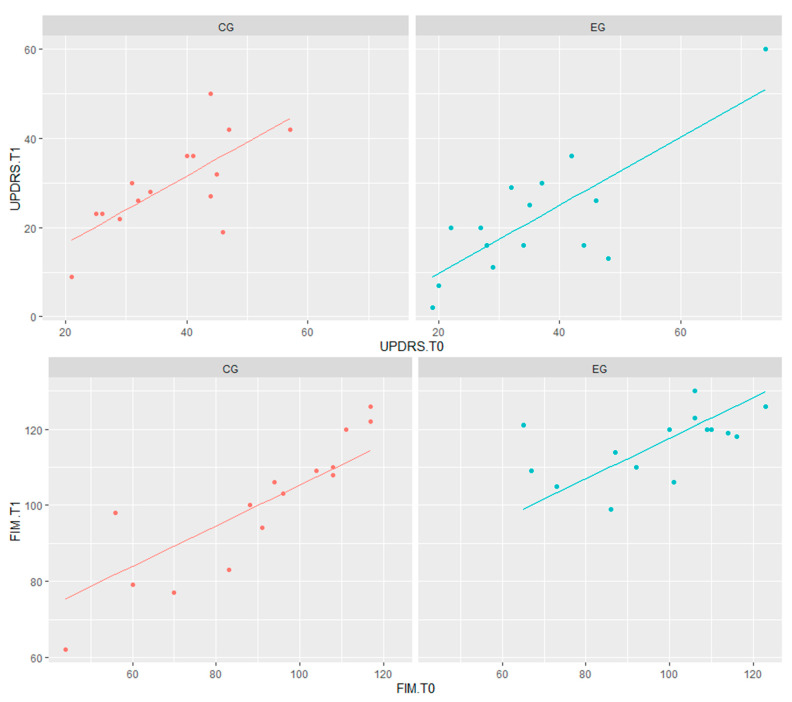
Graphical representation of ANCOVA analysis for UPDRS and FIM scores. Legend: CG = control group (in red); EG = experimental group (in blue); UPDRS = Unified Parkinson’s Disease Rating Scale; FIM = Functional Independence Measure.

Notably, the magnitude of improvement was greater for the EG. This was especially evident when comparing the changes in the UPDRS (*p* = 0.019) and FIM (*p* = 0.031) T1-T0 scores (see Figure 3). Something similar was seen for BBS, although it did not reach statistical significance (*p* = 0.095).

## 4. Discussion

To the best of our knowledge, this is the first study to explore the role of Rysen in promoting balance recovery and motor function in patients with PD. In our study, both groups (EG and CG) achieved clinical improvements, although the EG showed better post-treatment scores regarding the functional status (tested with the FIM) and global motor skills, including postural stability (measured with the MDS-UPDRS, section III), than the CG. Moreover, patients in the EG had improved BBS scores, reflecting a better balance performance, although this did not reach statistical significance. These findings could be explained by the fact that the BWS provided by the Rysen system allowed patients to walk with sustained trunk stability, ensuring a safe environment without the risk of falling [21]. In fact, PD patients may exhibit difficulties in assessing proper movement distances and an altered perception of external spaces and the environment due to a deficit in the integration of sensory inputs [22]. In this sense, the Rysen system may have contributed to a better improvement to postural control (PC). PC consists of maintaining body posture throughout space and ensuring balance against gravity and the position/orientation of the segments during movements [23]. Among the systems (i.e., visual, vestibular, and somatosensory) that are closely related to PC, the vestibular one controls the body’s centre of mass, both for static and dynamic positions, via postural responses, and it also stabilises the head during movements [24]. Patients affected by PD are more likely to develop alterations in vestibular and auditory pathways, as confirmed by Ampar et al. [25]. It has been shown that vestibular alterations are involved in the complex pathophysiological mechanisms subtending PI onset [25,26,27]. Indeed, vestibular information is transmitted to the basal ganglia, especially the striatum, whose dopaminergic input is affected by the disease [28]. This may result in the dysfunction of the pathway involving the pedunculopontine tegmental nucleus and the thalamus, and their connections with the substantia nigra and striatum, and this is responsible for the vestibulo-ocular and spinal reflexes [26]. 

Moreover, rhythmic lower limb movements and sensory inputs in the lower limbs could contribute to motor learning [29]. In this sense, the BWS system could activate the central pattern generator (CPG) in the spinal cord through rhythmic and cyclic gait movements, thus promoting a faster gait and an increased hip extension [30]. The CPG further allows the compensatory cortical excitation of supplementary motor and premotor areas, boosting the functional reorganization of neuronal circuits [31]. 

Furthermore, the effectiveness of the EG training session could be further exploited by the presence of audio-visual cues, placed in the patient’s surroundings to enhance motor skills [32]. In fact, sensory cues can activate the dorsolateral premotor control system, thus compensating for the deficiency in the supplementary motor area. Notably, it has been demonstrated that auditory cues could be helpful for improving the temporal parameters of gait (i.e., cadence and speed), while visual cues are more likely to improve spatial parameters (i.e., stride and step length) [33].

Recently, Lorenzo-Garcia et al. [14] carried out a systemic review with a metanalysis to determine the effectiveness of BWS gait training in PD patients. The authors found that BWS systems have a beneficial role in improving motor skills as per the UPDRS III, and they also promote stride length and balance. Nonetheless, there is not sufficient clinical evidence to confirm that BWS is useful for ameliorating cadence, gait speed, and the 6 min walking test score, according to the authors’ findings. Moreover, Berra et al. [34] compared the effects of BWS treadmill training with conventional overground gait training in patients with PD. The results found by these authors suggested that BWS treadmill training improved global motor skills and functioning (as per the FIM and UPDRS), confirming our results. However, they suggested that both types of training can be considered effective at inducing improvements in kinematic gait parameters. 

It is noteworthy that the Rysen system is quite different from traditional BWS systems because it provides not only vertical support, but also horizontal assistance, which is fundamental to compensating medio-lateral instability [13]. In fact, lateral falls are associated with a high risk of hip fractures, especially in old subjects and in patients with neurological disorders [35]. In this vein, the Rysen system could be a valid tool for fall prevention during the rehabilitation path. Additionally, thanks to this BWS system, the patient can move in each direction, involving the trunk in continuous adjustments to avoid the typical gait alterations in PD patients, such as the loss of the centre of mass and festination [14,34]. This may partly explain the improvement in the UPDRS score that we found in our EG. 

This study has some limitations that need to be stated. Firstly, the sample size was relatively small, and this is why our results cannot be generalized to the entire PD patient population. In addition, our results showed a significant statistical difference at the beginning of the study for the POMA, indicating that our groups were not perfectly homogeneous. Nonetheless, we performed the analysis of covariance (ANCOVA), which allowed us to assess whether the type of treatment influenced the clinical outcome independently from the score difference at baseline. Finally, we lacked a long-term follow-up and are unaware of whether and to what extent the effects of this intervention may last over time. Moreover, it could be helpful, in future larger-sample studies, to investigate the long-term effects of Rysen using a baropodometric evaluation and/or wearable sensors. 

## 5. Conclusions

In conclusion, our results suggest that the Rysen device can be considered a useful tool for improving postural control and global motor functions in patients affected by PD, although the data must be interpreted with caution given the limitations of the study. This system allows static and dynamic balance training, ensuring a patient’s sense of security and promoting a better quality of movement of the lower limbs in a safer rehabilitation setting. Since this is the first study about the role of Rysen in the context of PD rehabilitation, further studies with larger sample sizes and that use gait analysis information to objectively assess gait improvements are needed to confirm our promising results.

## Figures and Tables

**Table 1:** Clinical–demographic characteristics of the sample.

	All	EG	CG	*p*-Value
Participants: N	30	15	15	-
Male: N (%)	15 (50.0)	10 (66.7)	5 (33.3)	0.14
Female: N (%)	15 (50.0)	5 (33.3)	10 (66.7)	0.14
Age, years: median (IR)	68 (9.5)	68 (8.5)	68 (8.5)	0.85
DD, years: median (IR)	11.5 (8.75)	8 (9.5)	13 (7.5)	0.72
H&Y: median (IR)	3.5 (1)	3 (1)	3.5 (0.7)	0.23

Legend: EG = experimental group; CG = control group; DD = disease duration; IR = interquartile range; H&Y = Hoehn and Yahr score.

**Table 2:** Description of EG and CG exercise training.

Time	Objective	EG Exercise Training	CG Exercise Training
5’	Postural stability	Postural alignment with BWS	Postural alignment with the aid of a therapist
15’	Specific gait training	Forward/sideways/backward stepping at different speeds, with frequent and rapid changes in direction, while performing a dual-task activity to train shared attention (i.e., walking and counting backwards; walking and throwing a ball in the air), or walking along a path on surfaces of different textures (foam rubber mats, sandbags, wooden tablets, etc.), with the support provided by the Rysen system in addition to the supervision of a therapist.	Exercises for gait initiation comprised: weight shifting between lower limbs, stepping training over levels, heel strike/limb-loading acceptance, and push-off/initial swing of the moving limb. Overground gait exercises included: walking over obstacles with different sizes and colours and walking over different surfaces (e.g., foam rubber mats, sandbags, wooden tablets, etc.), always with the assistance of the physiotherapist.
15’	Static and dynamic balance	Static balance activities included: standing with a decreased base of support or on an unstable support surface, controlling heel–toe imbalances, and shifting the centre of gravity. Dynamic balance activities included: walking with frequent stops and changes in direction, and postural variations. Static–dynamic balance activities included: walking while holding a ball, a plate, or a tower of glasses in the palm of the hand during stable balance conditions. All these exercises were performed with the BWS system provided by Rysen, in addition to the supervision of a therapist.	Static balance activities included: standing with different variations in the base of support, tandem standing, and shifting the centre of gravity using an oscillating platform. Dynamic balance activities included: standing up and sitting down from a chair with or without using hands, walking in tandem, lateral weight shifting, stationary stepping, walking with frequent stops and changes in direction, and postural variations. All these exercises were performed with the continuous manual and visual assistance of a therapist.
5’	Return to the sitting position	Sit-down exercise was used in order to return to the sitting position, with the assistance of the Rysen system.	Sit-to-stand exercise and squats were used in order to return to the sitting position with the manual assistance provided by a therapist.

**Table 3:** ANCOVA results for each covariance model.

Clinical Assessment	Group Coefficient				Adjusted R^2^
	Estimate	Std. Error	*t*-Value	*p*-Value	
BBS	3.60	2.19	1.64	0.112	0.70
FES-I	−5.46	3.21	−1.70	0.101	0.14
POMA	1.32	1.88	0.70	0.487	0.48
UPDRS	−4.66	2.12	−2.20	0.036	0.59
FIM	12.36	3.36	3.67	0.001	0.68

Significant differences between treatment effects are in bold. Legend: BBS = Berg Balance Scale; FES-I = Falls Efficacy Scale—International; POMA = Tinetti’s Performance-Oriented Mobility Assessment; UPDRS-III = Unified Parkinson’s Disease Rating Scale; FIM = Functional Independence Measure.

## Data Availability

Data will be available on-demand from the corresponding author.
